# Influência da Atividade Física na Hipertensão Arterial em Trabalhadores

**DOI:** 10.36660/abc.20200318

**Published:** 2020-05-22

**Authors:** Cláudio L. Pereira da Cunha

**Affiliations:** 1 Universidade Federal do Paraná CuritibaPR Brasil Universidade Federal do Paraná (UFPR),Curitiba, PR – Brasil

**Keywords:** Hipertensão, Exercício, Fatores de Risco, Atividade Física, Epidemiologia, Trabalhadores, Limpeza Urbana

A atividade física (AF) regular é um dos mais importantes fatores para a prevenção primária da hipertensão arterial (HA) e para melhora da sobrevida a longo prazo desses pacientes.^1^ Seus benefícios se espalham para além da HA, propiciando melhoras também aos portadores de doença arterial coronariana, diabetes, dislipidemias, disfunção renal, depressão, doença pulmonar obstrutiva, osteoartrite, entre outras.^1^

“Atividade física”, de modo literal, se refere a qualquer movimento do corpo; no entanto, em estudos epidemiológicos que avaliam seu contexto na saúde, tem relação com atividades que produzam aumentos substanciais do consumo de oxigênio (O_2_). A AF pode ser realizada tanto durante o trabalho quanto no lazer e recreação, sendo todas as modalidades utilizadas no estudo dos efeitos da AF na saúde cardiovascular.^2^ A AF desenvolvida para melhorar a saúde ou obter benefícios no desempenho é considerada “exercício”.^2^

Os exercícios são classificados em “aeróbicos” e “resistivos” (ou de resistência). Os exercícios aeróbicos primariamente estressam o sistema de transporte de O_2_ e incluem atividades como caminhadas, corridas, natação e ciclismo. Os exercícios de resistência, por sua vez, estressam o sistema muscular esquelético, como o levantamento de peso; podem ser resistivos dinâmicos ou estáticos (isométricos). O treinamento com exercícios realizados repetidamente podem aumentar o desempenho do sistema cardiovascular (treinamento com exercícios aeróbicos) ou do sistema muscular esquelético (treinamento com exercícios resistivos).^3^ Tais divisões dos tipos de AF são relativamente arbitrárias, visto que os exercícios têm componentes aeróbicos ou resistivos predominantes mas não absolutos. A atividade aeróbica da corrida, por exemplo, também provoca melhora da força muscular das pernas, enquanto o exercício resistivo de levantamento de peso também cursa com envolvimento do sistema de entrega do O_2_, um componente aeróbico.^4^

O treinamento aeróbico reduz a pressão arterial (PA) casual de pré-hipertensos e hipertensos. Além disso, reduz a PA na vigília de hipertensos e diminui a PA em situações de estresse físico, mental e psicológico.^5^ O treinamento aeróbico é recomendado como forma preferencial de exercício para a prevenção e tratamento da HA, com grau de recomendação I, nível de evidência A, na 7ª Diretriz Brasileira de Hipertensão Arterial.^5^ Além da aptidão aeróbica, outros componentes da aptidão física estão associados ao prognóstico; estudos com força e potência muscular também têm demonstrado associações à mortalidade. As diretrizes recomendam a prática regular e combinada dos exercícios aeróbicos e resistidos^6,7^

A AF pode ser realizada no trabalho, no seu deslocamento, em trabalhos domésticos ou em práticas físicas de lazer.^8^Não há consenso sobre o benefício à saúde que cada uma dessas modalidades de AF pode propiciar, qual a contribuição que cada tipo pode oferecer ou até mesmo quais prejuízos podem determinar.^8,9^

Nesta edição dos *Arquivos Brasileiros de Cardiologia*, Ribeiro Jr. e Fernandes^10^ analisam o efeito cumulativo de diferentes modalidades de AF na HA de trabalhadores. Estudam 1.070 participantes vinculados a 2 empresas com características de exigência física bem distintas: 624 trabalhadores em limpeza urbana e 446 em indústria de calçados. Em todos os trabalhadores é avaliada sua AF em diferentes modalidades: ocupacional (AFO), doméstica (AFD) e de lazer (AFL). Analisa-se a ocorrência de HA, considerando como principal variável o número de modalidades de AF que o trabalhador executava (AFO, AFD e/ou AFL), e conclui-se que há efeito cumulativo das diversas formas de AF na proteção à HA.

Nesta pesquisa^10^são incluídas classes profissionais que caracterizam muito bem comportamentos opostos em relação ao grau de AFO, envolvendo grande número de trabalhadores braçais da limpeza urbana e outros pouco ativos na indústria manufatureira convencional. Todavia, não se avaliou a AF de deslocamento, mais facilmente analisável e frequentemente valorizada em tais estudos,^8^ e incluiu-se a AFD, presente no mundo real, mas com quantificação mais difícil, notadamente nesta amostra populacional com quase 80% do sexo masculino. Na análise multivariada, a AF foi avaliada em suas três modalidades (AFO, AFD e ADL), e os trabalhadores ativos nos três tipos de AF compuseram o primeiro grupo, os ativos em duas modalidades formaram outro grupo e, no terceiro, ficaram os trabalhadores inativos ou ativos em apenas um tipo de AF. Essa divisão é passível de críticas: por exemplo, não impossível no mundo real, um empregado da fábrica de calçados (pouca AFO), pouco envolvido em AFD, mas que faz 1 hora de exercícios 5 vezes na semana (300 minutos na semana), seria classificado para o terceiro grupo (ativo em apenas uma modalidade de AF, junto com os inativos).^10^

A contribuição da AFO no controle da HA é defendida pela maioria dos estudiosos,^2,8,11^mas não é uma opinião consensual.^9,12,13^Vários estudos epidemiológicos têm mostrado que o risco para doença cardiovascular e morte pode aumentar com AFO.^12^Esses efeitos contrastantes da AF na saúde têm sido chamados de “paradoxo da atividade física na saúde”.^9^O risco aumentado na AFO tem sido mostrado com maior frequência em trabalhadores com baixa renda, baixa aptidão cardiorrespiratória e doenças preexistentes, como HA e doença coronariana.^13^ Holtermann et al.^9^indicam que algumas diferenças entre AFL e AFO podem justificar a diferença dos resultados observados, incluindo: 1) AFO com intensidade muito baixa e duração muito longa para melhorar a forma física e a saúde cardiovascular; 2) AFO com levantamento de pesos ou posturas estáticas, que podem aumentar a PA; 3) AFO realizada sem tempo de recuperação suficiente; 4) AFO realizada com pouco controle pelo trabalhador; e 5) AFO que aumenta os níveis de inflamação. O tipo errado de AF pode ser ruim para a saúde, tanto no contexto de AFO como AFL.^13^Assim, muita força mecânica pode causar uma lesão musculoesquelética, atividade muito frequente pode levar à fadiga e muito tempo em pé pode facilitar o aparecimento de varizes nos membros inferiores. Ao contrário, muito pouca força pode causar perda óssea e muscular, exercícios pouco frequentes podem determinar um descondicionamento cardiorrespiratório ou alteração da saúde cardiometabólica.^13^Assim, os benefícios da atividade física, tanto no trabalho como no lazer, só se manifestam quando os vários aspectos da AF estão bem ajustados, calibrados. As várias dimensões da AF (intensidade, duração, frequência de posturas e movimentos distintos) afetam diferentes sistemas e funções do corpo (capacidade aeróbica, força muscular, movimentos, balanço, coordenação). Todos esses aspectos da AF no trabalho deverão levar ao encontro de seu “ponto perfeito”, para que venham a permitir o seu efeito de promover saúde.^13^

Em conclusão, a AF é benéfica à saúde e ajuda a controlar a PA, principalmente com a prática de exercícios aeróbicos, mas também com os exercícios resistivos. O efeito cumulativo de diferentes modalidades da AF, como estudado por Ribeiro Jr. & Fernandes,^10^ contribui para a proteção à HA. A AFO deve ser avaliada com cautela e profundidade, de maneira a se encontrar situações ótimas das atividades físicas, de forma a utilizá-la em benefício da saúde, e não como um mecanismo deletério para o trabalhador.
