# Supplementary material for: Influência da Atividade Física na Hipertensão Arterial em Trabalhadores
Source: Arq Bras Cardiol. 2020 May 22;114(5):762–3. [Article in Portuguese] doi: 10.36660/abc.20200318 (PMC8387001; doi:10.36660/abc.20200318)
Supplement: Translation

# Translation: Influence of Physical Activity on Arterial Hypertension in Workers

**Authors:** Cláudio L. Pereira da Cunha

**Affiliations:** Brazil Universidade Federal do Paraná (UFPR),1 Curitiba, PR – Brazil

**Keywords:** Hypertension, Exercise, Risk Factors, Physical Activity, Epidemiology, Workers, Urban Cleaning

Regular physical activity (PA) is one of the most important factors for the primary prevention of arterial hypertension (AH) and for improving the long-term survival of these patients.^1^ Its benefits extend beyond AH, also providing improvements to patients with coronary artery disease, diabetes, dyslipidemia, kidney dysfunction, depression, obstructive pulmonary disease, and osteoarthritis, among others.^1^

“Physical activity” literally refers to any movement of the body; however, in epidemiological studies that assess its health context, it is related to activities that produce substantial increases in oxygen (O_2_) consumption. PA can be performed both at work and during leisure and recreation, with all modalities being used to study the effects of PA on cardiovascular health.^2^ The PA developed to improve health or to obtain performance benefits is considered “exercise”.^2^

The exercises are classified as “aerobic” and “resistance” exercises. Aerobic exercises primarily stress the O_2_ transportation system and include activities such as walking, running, swimming and cycling. Resistance exercises, in turn, stress the muscular skeletal system, such as weightlifting; they can be dynamic or static exercises (isometric). Training with exercises that are performed repeatedly can improve the cardiovascular system performance (training with aerobic exercises) or the muscular skeletal system (training with resistance exercises).^3^ Such divisions of the types of PA are relatively arbitrary, considering the exercises have predominant but not absolute aerobic or resistance components. The aerobic activity of running, for instance, also improves the muscular strength of the legs, while the resistance weightlifting exercise also involves the participation of the O_2_ delivery system, an aerobic component.^4^

The aerobic training reduces the casual blood pressure (BP) in pre-hypertensive and hypertensive individuals. Moreover, it reduces BP during wakefulness in hypertensive patients and decreases BP in situations of physical, mental and psychological stress.^5^ Aerobic training is recommended as the preferred form of exercise for the prevention and treatment of AH, with a class of recommendation I, level of evidence A, in the 7^th^ Brazilian Guideline of Arterial Hypertension.^5^ In addition to aerobic fitness, other components of physical fitness are associated with the prognosis; studies with muscle strength and power have also shown associations with mortality. The guidelines recommend the regular and combined practice of aerobic and resistance exercises.^6,7^

The PA can be performed at work, in displacement, during housework or during leisure practices.^8^ There is no consensus about the health benefits that each of these types of PA can provide, what contribution each type can offer or even what harms they can determine.^8,9^

In this issue of the *Arquivos Brasileiros de Cardiologia*, Ribeiro Jr. and Fernandes^10^ analyze the cumulative effect of different types of PA on the workers’ AH. They studied 1,070 participants who worked for two companies with quite different physical requirements: 624 workers in urban cleaning and 446 in the shoe industry. The PA was evaluated in all workers in the different modalities: occupational (OPA), domestic (DPA) and leisure (LPA). The occurrence of AH was analyzed considering as the main variable the number of PA modalities that the worker performed (OPA, DPA and/or LPA), and it was concluded that there was a cumulative effect of the different types of PA in the protection against AH.

This research^10^ includes professional classes that characterize very well opposite behaviors in relation to the degree of OPA, involving a large number of manual workers employed in urban cleaning and others who are not very active in the conventional manufacturing industry. However, PA in displacement, which is more easily analyzed and frequently estimated in such studies,^8^ was not evaluated, and DPA, present in the real world but more difficult to quantify, was included, notably in this population sample with almost 80% of male individuals. In the multivariate analysis, PA was evaluated in the three modalities (OPA, DPA and LPA), and the workers active in the three types of PA comprised the first group, those active in two modalities comprised another group and, the third consisted of workers who were inactive or active in only one type of PA. This division is liable to criticism: for instance, not impossible in the real world, an employee of the shoe factory (little OPA), little involved in DPA, but who performs 1 hour of exercise 5 times a week (300 minutes a week), would be classified as belonging to the third group (active in only one PA modality, together with the inactive ones).^10^

The contribution of OPA to AH control is defended by most researchers,^2,8,11^ but it is not a consensus opinion.^9,12,13^ Several epidemiological studies have shown that the risk of cardiovascular disease and death can increase with OPA.^12^ These contrasting effects of PA on health have been called the “physical activity paradox” in health.^9^The increased risk in OPA has been demonstrated more frequently in workers with low income, low cardiorespiratory fitness and pre-existing diseases, such as AH and coronary disease.^13^ Holtermann et al.^9^ indicate that some differences between LPA and OPA may justify the difference in the observed results, including: 1) OPA with very low intensity and very long duration to improve physical fitness and cardiovascular health; 2) OPA with weightlifting or static postures, which can increase BP; 3) OPA performed without sufficient recovery time; 4) OPA performed with little control by the worker; and 5) OPA that increases inflammation levels. The wrong type of PA can be harmful to health, both in the context of OPA and LPA.^13^ Therefore, too much mechanical force can cause a musculoskeletal injury, a too frequent activity can lead to exhaustion and too much time in the standing position can facilitate the appearance of varicose veins in the lower limbs. In contrast, too little strength can cause bone and muscle loss, infrequent exercises can cause cardiorespiratory deconditioning or cardiometabolic health alteration.^13^Thus, the benefits of physical activity, both at work and during leisure, are only manifested when the several aspects of PA are well adjusted, calibrated. The different dimensions of PA (intensity, duration, frequency of postures and different movements) affect different body systems and functions (aerobic capacity, muscle strength, movements, balance, coordination). All these aspects of PA at work should lead to the meeting of one’s “perfect point”, so that their effect in health promotion can occur.^13^

In conclusion, PA is beneficial to health and helps to control BP, mainly through the practice of aerobic exercises, but also with resistance exercises. The cumulative effect of different types of PA, as studied by Ribeiro Jr. & Fernandes,^10^ contributes to the protection against AH. The OPA should be evaluated carefully and in details, aiming to find optimal physical activity situations, so they can be used for health benefits, and not as a harmful mechanism for the worker.
